# Eigenvector centrality defines hierarchy and predicts graduation in therapeutic community units

**DOI:** 10.1371/journal.pone.0261405

**Published:** 2021-12-16

**Authors:** Benjamin Campbell, Keith Warren, Mackenzie Weiler, George De Leon

**Affiliations:** 1 Department of Political Science, The Ohio State University, Columbus, Ohio, United States of America; 2 The Ohio State University College of Social Work, Columbus, Ohio, United States of America; 3 New York University Rory Meyers College of Nursing, New York, New York, United States of America; University of Padua, ITALY

## Abstract

**Introduction:**

Therapeutic communities (TCs) are mutual aid based residential programs for the treatment of substance abuse and criminal behavior. While it is expected that residents will provide feedback to peers, there has been no social network study of the hierarchy through which feedback flows.

**Methods:**

Data for this study was drawn from clinical records of peer corrections exchanged between TC residents in six units kept over periods of less than two to over eight years. Four of the units served men while two served women. Hierarchy position was measured using eigenvector centrality, on the assumption that residents who were more central in the network of corrections were lower in the hierarchy. It was hypothesized that residents would rise in the hierarchy over time. This was tested using Wilcoxon paired samples tests comparing the mean and maximum eigenvector centrality for time in treatment with those in the last month of treatment. It was also hypothesized that residents who rose higher in the hierarchy were more likely to graduate, the outcome of primary interest. Logistic regression was used to test hierarchy position as a predictor of graduation, controlling for age, race, risk of recidivism as measured by the Level of Services Inventory-Revised (LSI-R) and days spent in the program.

**Results:**

Residents averaged a statistically significantly lower eigenvector centrality in the last month in all units, indicating a rise in the hierarchy over time. Residents with lower maximum and average eigenvector centrality both over the length of treatment and in the last month of treatment were more likely to graduate in four of the six units, those with lower maximum and average eigenvector centrality in the last month but not over the length of treatment were more likely to graduate in one of the six units, while eigenvector centrality did not predict graduation in one unit. However, this last unit was much smaller than the others, which may have influenced the results.

**Conclusion:**

These results suggest that TC residents move through a social network hierarchy and that movement through the hierarchy predicts successful graduation.

## Introduction

Therapeutic communities (TCs) are residential programs for substance abuse and criminal behavior that depend on mutual aid between residents as a core clinical methodology [1–4]. One aspect of mutual aid in TCs is that residents are expected to monitor each other’s behavior and offer feedback by either affirming prosocial behavior or correcting behavior that contravenes TC norms [1, 5, 6]. This system of feedback encourages changes in both behavior and social identity, from those associated with addiction to those associated with recovery [7–9].

It is not obvious how TCs manage to maintain a system that provides both orderly and clinically useful peer feedback. There is evidence that TC residents engage in reciprocal exchanges of feedback, as well as generalized reciprocity [10, 11]. TC residents tend to form closed triads and successful residents have somewhat more interaction with other successful residents [12]. There is also evidence that roommate relations and the assignment of big brothers or sisters when residents enter the program impact success [13]. However, these are local interactions, while TC clinical theory sees the community as a whole as being the method of treatment [1].

TC clinical theory also emphasizes the importance of senior residents acting as role models, suggesting that a hierarchy in which some residents garner more respect than others might play a role. Hierarchy is of particular interest in TCs because we would expect that those residents who have risen the highest in the hierarchy have also taken the most responsibility for peers and learned the most in the program. They should therefore be more likely to successfully graduate. Successful graduation, in turn, is the most important proximal outcome of treatment, because graduates tend to have better outcomes in the community [14, 15]. Several studies suggest that relationship with peers predicts successful graduation from TCs. Carr & Ball found that resident rating of the orderliness of the unit predicted retention in a TC [16]. Mandell et al. found that the Dimensions of Change Instrument (DCI), which includes measures of attitudes toward peers, predicted retention [17]. Taking a social network approach, Campbell et al. [18] and Warren et al. [19] have found that residents who graduated were more likely to interact with peers who also graduate.

There have been several studies that seek to define and evaluate the TC hierarchy. In a study of a prison-based TC, Patenaude [6] found that TC residents often resented the hierarchical structure, arguing that more senior members of the TC who were higher in the hierarchy were criminals as well and had no right to pass judgment. In a case study of a single resident of a correctional TC, Greenall [20] found the hierarchy to be of value. Toumbourou et al. [21] found that residents who attained a higher level of program treatment, who might be assumed to be higher in a resident hierarchy, had better post-treatment outcomes. At this point there has been no social network analysis of hierarchy in TCs and there has been no study of whether progress through the hierarchy of TC residents predicts graduation.

Beyond TCs, there have been network based studies of hierarchy in both human and biological social systems. From the study of world politics [22] to university professors [23], scholars have detected clear hierarchical relations in networks of social relations. In a social network study of Oxford Houses, small scale mutual aid based programs for individuals with substance abuse, Doogan et al. [24] found that residents with better self-rated quality of life were likely to connect with peers with worse self-rated quality of life, suggesting a hierarchy in which stronger residents offer help to weaker housemates. Hierarchical structure has also been found in groups of animals. For instance, Hobson and DeDeo [25] find that hierarchy is an emergent phenomena of parakeet interactions, whereby parakeets learn their position in the hierarchy as a function of who aggresses against whom.

In this study we analyze hierarchy in six units in three TCs and the relationship of movement in that hierarchy to successful graduation. The data which we analyze is a large network composed of peer corrections exchanged between TC residents. Corrective peer feedback is common in TCs, used both for maintaining standards of behavior within programs and encouraging residents to learn new patterns of behavior [1, 2].

Following the precedent of previous studies [25–27] we use eigenvector centrality calculated on the network of therapeutic community corrections to infer the unit’s hierarchy. While there are a number of different centrality measures [28], this approach allows us to infer an individual’s position in the hierarchy based not just on who corrects them, but who corrects the peers that correct them. As residents’ positions in the network of corrections change over time their position in the hierarchy changes. We expect that residents will on average rise in the hierarchy over the course of treatment. We find support for this hypothesis across all TC units. We also expect that those residents who rise in the hierarchy are more likely to graduate successfully from the program, when controlling for time in treatment. We find support for this hypothesis across five of six TC units: position in the TC hierarchy reveals important information about a resident’s likelihood of graduation. We do not receive support for a third hypothesis, that the relationship between position in the hierarchy during the last month will be a stronger predictor of graduation than position in the hierarchy during previous months.

## Methodology

### Setting

This project was declared to not be human subjects research on 5/11/2018 by The Ohio State University Office of Responsible Research on the grounds that the data is a de-identified database that was originally gathered for clinical purposes. The data for this study was drawn from three Midwestern free standing community based correctional facilities run as TCs in a Midwestern state. Descriptive figures for the facilities can be found in Table 1. Facility 1 was the largest of the three, and served a mixed urban and rural catchment area of six counties. Men in Facility 1 lived in two units with eighty beds each and women lived in one unit with eighty beds. Each of the male units included four dormitories for sleeping and a common area in which all men in the unit interacted during the day. The female unit consisted of six dormitories for sleeping and a common area in which all women in the unit interacted during the day. Facility 2 was a male-only TC serving a rural catchment area of five counties with one sixty-four bed unit. The unit consisted of four dormitories for sleeping and one common room in which the residents interacted during the day. Facility 3 served a rural and suburban catchment area of eight counties. The male unit had a total of ninety beds divided between four dormitories and a common area. The female unit had one sixteen bed dormitory. The female unit in Facility 3 was also started a number of years after the male unit.

**Table 1:** Descriptive information for units.

Facility/Unit	Number of Counties in catchment area	Rural, suburban or urban	Number of Beds	Number of Dormitories
Facility 1/Men’s unit 1	6	Urban, rural and suburban	80	4
Facility 1/Men’s unit 1	6	Urban, rural and suburban	80	4
Facility 1/Women’s unit	6	Urban, rural and suburban	80	6
Facility 2 (Men only)	5	Rural	64	4
Facility 3/Men’s unit	8	Rural, suburban	90	4
Facility 3/Women’s unit	8	Rural, suburban	16	1

### Data

The structured nature of TCs offers an advantage in measuring hierarchy, since it is assumed that residents will offer feedback to peers, correcting any behaviors that contravene TC norms [1, 2, 29]. Over time these corrections form a longitudinal directed network in which each node is an individual resident, while each edge is a correction that a resident sent to a peer. Thus, if Resident A corrects Resident B it establishes a directed edge between the two. For this analysis we aggregated daily data over a series of one week time periods so as to eliminate any effect of weekly periodicity.

**Fig 1:**
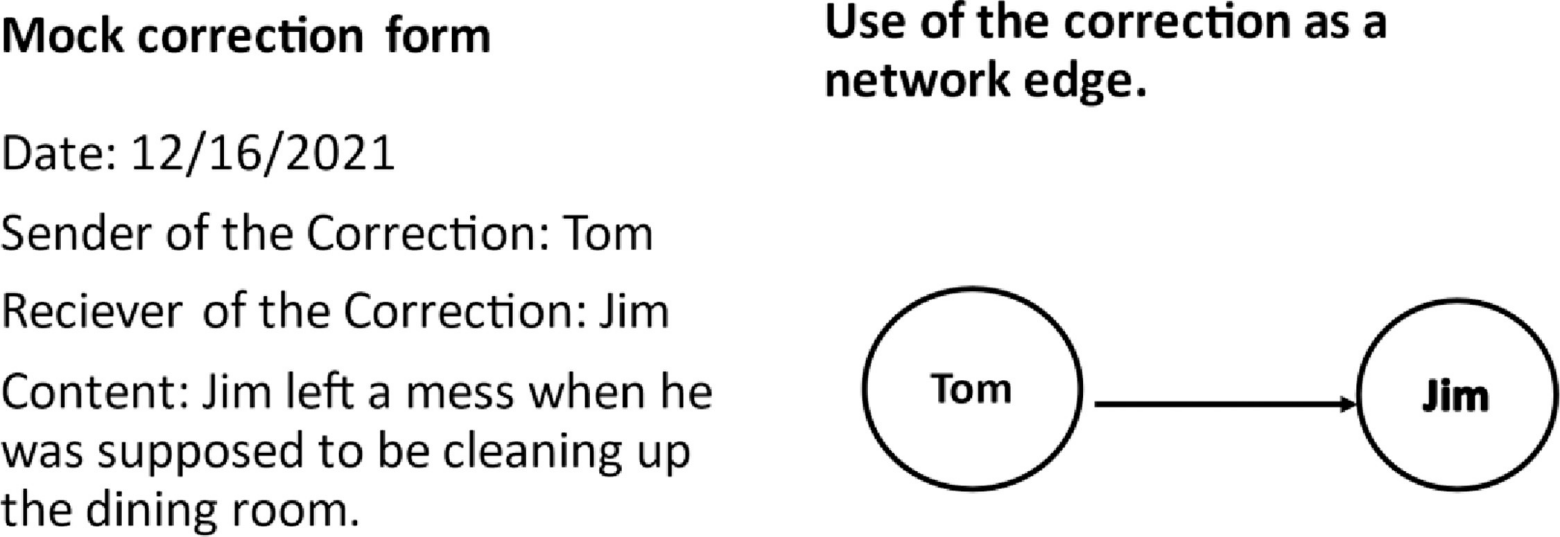
A mock correction form and network edge.

The corrections recorded in this study were kept as archival data for the purposes of monitoring the clinical functioning of six TC units. The person who wanted to send a correction would first write it down on a form that included the date, the name of the sender, the name of the person receiving the correction and the content of the correction. A committee of senior residents and staff would then review the form for legitimacy; for instance, there was a conscious effort to assure that corrections were not simply given in revenge for previous corrections. Once they had been reviewed, they were announced at a meeting of the entire TC. They were then entered into an electronic database which was used for monitoring activity on the units. Fig 1 illustrates the relationship between a mock correction and a directed edge; the edge that results naturally does not include the actual content of the correction.

The programs also kept records of resident age, race, the date on which each resident entered and terminated from treatment, and residents’ scores on the Level of Service Inventory-Revised (LSI-R) [30], a well-validated and reliable instrument designed to predict risk of reincarceration using known factors including substance abuse history, criminal history, education and employment skills, family status, and leisure skills. These risk factors are associated with recidivism for both male and female offenders [31].

### Analysis

To detect and measure the hierarchy of social systems, researchers typically construct a directed social network wherein one actor relates to another in some way. For instance, researchers might treat answers to the question, “Who do you regard as a friend?” as a directed social network, with an arrow pointing from individual A to individual B if A states that he/she regards B as a friend. It is typically assumed that people who are more central in the network have higher status [32]. There are a number of ways to measure centrality in a directed network [28]. The simplest is indegree centrality, which is simply a count of the number of arrows pointing at any individual. In the example above indegree centrality would measure how many people name a given individual as a friend.

Anyone who has ever been part of a hierarchy will immediately spot the weakness of indegree centrality as a measure of position. It’s usually more valuable if the people who are friends with an individual themselves have many friends; one will be higher in the hierarchy if one is part of the in-crowd. Thus, an alternative measure of centrality, eigenvector centrality, is useful for studies of hierarchy. Eigenvector centrality weights peer connections by the number of connections that those peers have. Effectively, you’re higher in the hierarchy if your friends have lots of friends [33].

**Fig 2:**
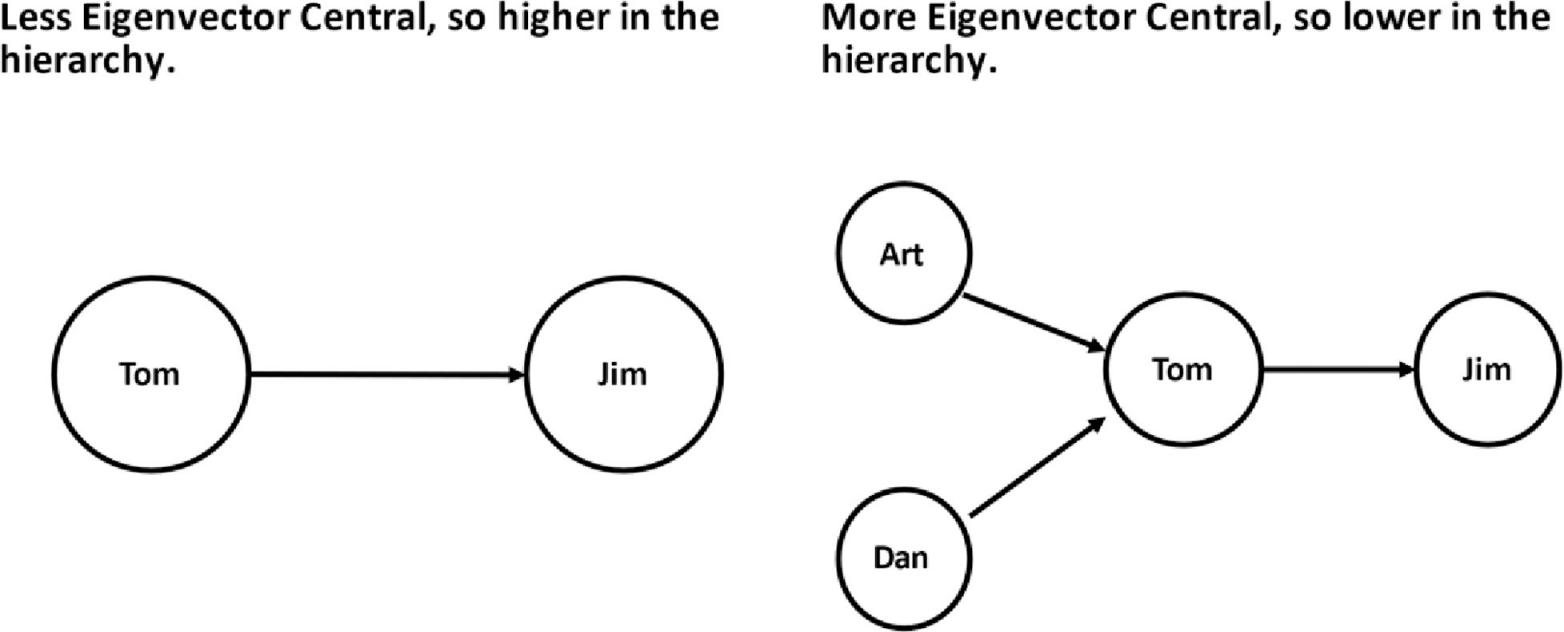
Jim’s place in the hierarchy as measured by eigenvector centrality depends on who has corrected Tom.

Within the context of studying hierarchy using negatively valenced relations the logic is reversed [25–27, 32–34]. Instead of implying influence and power, higher eigenvector centralities imply a lower position in the hierarchy. In the animal kingdom, Bird A may be pecked by Bird B, who is in turn pecked by Birds C and D. In this situation, bird A has the highest eigenvector centrality since bird B, who is pecking A, is being pecked by the two birds C and D, who are not themselves being pecked at all [25]. Bird A also is clearly the lowest member of the hierarchical pecking order. While corrections in TCs are intended to help peers rather than exert dominance, the logic is the same. Residents who receive corrections from individuals who themselves receive many corrections would be considered to occupy low positions in the hierarchy, while those who send corrections but receive few or none would be considered to occupy high positions in the hierarchy. Fig 2 gives a simple example of this relationship.

An individual is therefore moving up in the TC hierarchy if his or her eigenvector centrality is decreasing over time. Those individuals who attain the lowest eigenvector centrality have attained the highest position in the hierarchy.

In the context of hierarchy based on a negatively valenced network connection, eigenvector centrality maintains its value as a measure of hierarchy because it takes into account the higher order network dependencies that most accurately capture hierarchy as an emergent phenomenon. In contrast, indegree centrality treats all alters as equivalent which intrinsically implies that receiving a correction from someone who never receives corrections is the same as receiving a correction from someone who receives many of them. While other measures, such as betweenness centrality and closeness centrality, are useful for measuring information flow in networks [28], only eigenvector centrality captures this particular dynamic that is essential to measuring hierarchy [25–27, 32–34]. Given their inability to measure hierarchy, our concept of interest, we do not examine the relationship between these other inapplicable measures and graduation.

It would also be possible to rely upon a measure of seniority, such as time in the facility, as a proxy for position in the TC hierarchy. But this approach would make the explicit assumption that position in hierarchy is only a function of time. This is clearly problematic as senior members of the TC may have different responses to treatment. Measurements computed on the network are therefore critical as the network is the manifestation of the latent hierarchy. Moreover, the correlation between time in treatment and graduation approaches the tautological, since successful graduates have by definition not been prematurely terminated.

In this analysis we measure each individual’s position in the hierarchy in four ways. Maximum Eigenvector Centrality is defined as an individual’s maximum eigenvector centrality over their full course of treatment, which is designed to capture the lowest position they may ever achieve. Mean Eigenvector Centrality is the average of an individual’s eigenvector centrality over their full course of treatment. Our theory would dictate that this should matter more than Maximum Eigenvector Centrality in predicting successful graduation from a TC, but not as much as other measures which capture how well someone has responded to treatment towards the end of their time on the unit. Last Month Maximum Eigenvector Centrality is the same as Maximum Eigenvector Centrality except it is only calculated over the last four weeks of their time at the TC, instead of over their full time in the TC. The same is true for Last Month Mean Eigenvector Centrality compared to Mean Eigenvector Centrality.

These measurements yield three hypotheses. First, we would expect that residents’ Last Month Maximum Eigenvector Centrality and Last Month Mean Eigenvector Centrality would be lower than their overall Maximum and Mean Eigenvector Centralities. This would be true if residents typically progressed through the TC hierarchy. Second, we would expect that all Eigenvector Centrality measures would be negatively correlated with the likelihood of graduation; residents with lower scores are more likely to graduate. Finally, we would expect the last month scores to be better predictors of graduation when measured by effect size.

We analyze the difference between mean and maximum Eigenvector Centrality for the entire period of treatment and those for the final month of treatment using a Wilcoxon signed rank test with a continuity correction [35]. We examine the relationship between the four measures of a resident’s position in the hierarchy and their propensity to graduate from their recovery program using logistic regression, while controlling for time in treatment, which would be expected to correlate with position in the hierarchy and is almost by definition longer on average for those who successfully graduate. We also control for age, score on the LSI-R and race.

## Results

Descriptive statistics can be found it Table 2. Graduation rates for all of these TCs are quite high, and the percentage of racial minority residents in Facility 2 and Facility 3 is substantially lower than is typical of the American prison system. As expected, in all cases both the mean eigenvector centrality and maximum eigenvector centrality for the last month is lower than those for treatment as a whole, suggesting that most residents do progress through the hierarchy.

**Table 2:** Descriptive statistics, with Wilcoxon signed rank test comparing overall eigenvector centrality with last month eigenvector centrality.

Variable	Facility 1, Women’s Unit	Facility 1, Men’s Unit 1	Facility 1, Men’s Unit 2	Facility 2, Men’s Unit	Facility 3, Women’s Unit	Facility 3, Men’s Unit
Graduation*	0.86	0.86	0.85	0.78	0.78	0.83
Age	31.04	28.06	30.91	27.10	32.10	27.87
	(8.37)	(8.62)	(9.38)	(8.84)	(7.33)	(8.62)
LSI	25.46	25.80	26.01	31.84	21.14	23.18
	(7.19)	(5.60)	(6.32)	(7.95)	(6.07)	(7.56)
Race*	0.21	0.49	0.24	0.09	0.07	0.15
Days in Program	135	117.40	139.20	162	140.10	146.50
	(34.41)	(33.17)	(36.28)	(38.13)	(45.02)	(33.35)
Maximum Eigenvector Centrality	0.63	0.23	0.16	0.50	0.81	0.53
	(0.26)	(0.27)	(0.24)	(0.40)	(0.28)	(0.30)
Last Month Maximum Eigenvector Centrality	0.35	0.07	0.05	0.25	0.55	0.28
	(0.25)	(0.15)	(0.16)	(0.34)	(0.32)	(0.27)
Wilcoxon Signed Rank Test	V = 420903	V = 179700	V = 35511	V = 162735	V = 1128	V = 687378
	p<2.2e-16	p<2.2e-16	p<2.2e-16	p<2.2e-16	p = 2.48e-09	p<2.2e-16
Mean Eigenvector Centrality	0.21	0.03	0.02	0.10	0.34	0.12
	(0.11)	(0.04)	(0.03)	(0.11)	(0.18)	(0.09)
Last Month Mean Eigenvector Centrality	0.17	0.02	0.01	0.08	0.27	0.11
	(0.14)	(0.05)	(0.04)	(0.12)	(0.19)	(0.12)
Wilcoxon Signed Rank Test	V = 466407	V = 234528	V = 45135	V = 227722	V = 1827	V = 779587
	p<2.2e-16	p<2.2e-16	p = 0.00026	p<2.36e-10	p = 4.11e-06	p<2.2e-16
Days of Observation	2844	2844	2844	2731	604	3244

* Dichotomous variables have only proportions provided. Means shown with standard deviations in parentheses.

**Fig 3:**
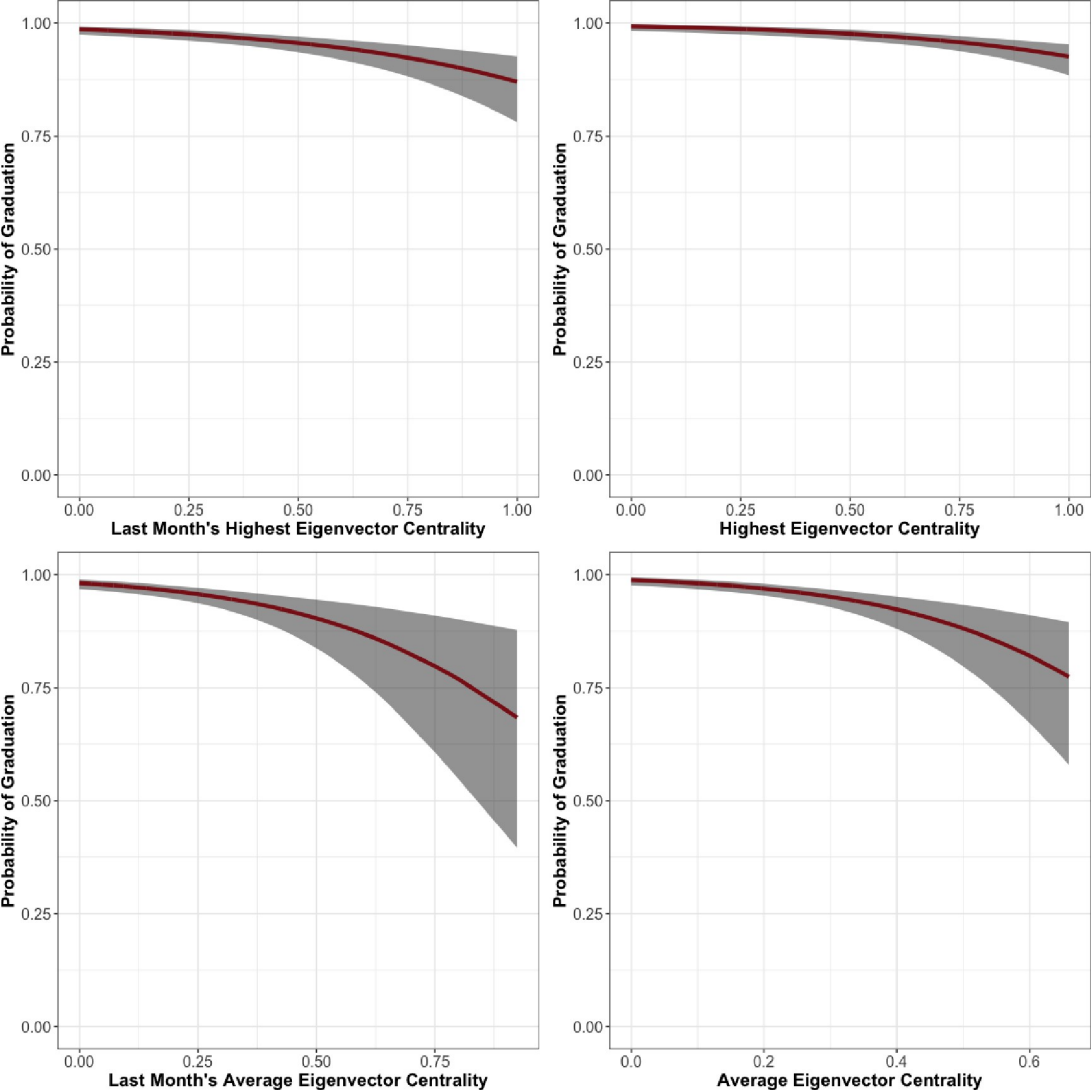
Facility 1, Women’s unit predicted probability of graduation by eigenvector centrality.

Results for the Facility 1 women’s unit are in Table 3. In this unit, we find that age does not correlate with successful graduation at any conventional threshold of statistical significance. Participants’ LSI-R, race, tenure in the program, and hierarchy are correlated with graduation at any conventional threshold for statistical significance. As a respondent’s LSI-R score increases, they are less likely to graduate. Black residents are also less likely to graduate, all other things being equal, than white residents. As a resident spends more time in treatment they are naturally more likely to graduate. Each measure of an individual’s position in the hierarchy is a statistically significant predictor of graduation. Last Month Maximum Eigenvector Centrality (*β* = -2.36, *se* = 0.47), Last Month Average Eigenvector Centrality (*β* = -3.46, *se* = 0.82), Maximum Eigenvector Centrality (*β* = -2.39, *se* = 0.53), and Average Eigenvector Centrality (*β* = -4.83, *se* = 1.06) were all significant in the expected negative direction. The relationships between eigenvector centrality over the entire length of treatment and during the last month and predicted probability of graduation are shown in Fig 3.

**Table 3:** Facility 1, Women’s unit: Eigenvector centrality as a predictor of graduation.

	Model 1	Model 2	Model 3	Model 4
Intercept	3.13**	3.02**	3.28**	3.16**
	(1.02)	(1.01)	(1.03)	(1.01)
Age	0.02	0.02	0.01	0.01
	(0.02)	(0.02)	(0.02)	(0.02)
LSI-R	-0.22***	-0.23***	-0.22***	-0.22***
	(0.02)	(0.02)	(0.02)	(0.02)
Race	-0.99***	-1.01***	-0.94***	-1.00***
	(0.29)	(0.29)	(0.29)	(0.29)
Days in Program	0.05***	0.04***	0.05***	0.05***
	(0.00)	(0.00)	(0.01)	(0.00)
Last Month Maximum Eigenvector Centrality	-2.36***			
	(0.47)			
Last Month Average Eigenvector Centrality		-3.46***		
		(0.82)		
Maximum Eigenvector Centrality			-2.39***	
			(0.53)	
Average Eigenvector Centrality				-4.83***
				(1.06)
AIC	458.40	465.68	461.62	462.31
BIC	488.27	495.72	491.49	492.18
Log-Likelihood	-223.20	-226.93	-224.81	-225.16
Deviance	446.40	453.86	449.62	450.31
N	1072	1072	1072	1072

*** p < 0.001,

** p < 0.01,

* p < 0.05.

**Fig 4:**
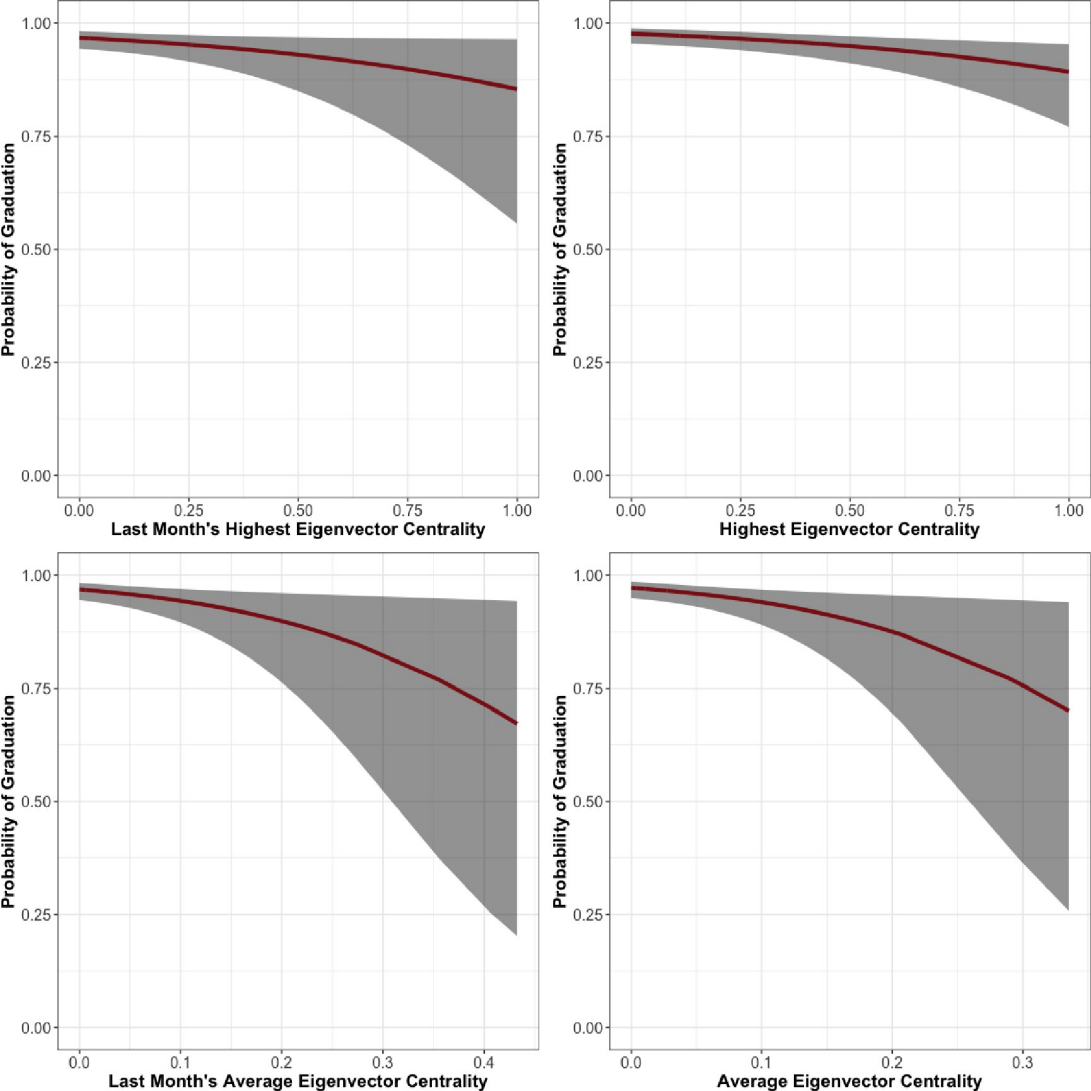
Facility 1, Men’s unit 1 predicted probability of graduation by eigenvector centrality.

In the first Men’s unit of Facility 1 in Table 4 we see similar relationships. LSI-R and the number of days in the program predict graduation in the expected direction with a robustness significant at any conventional threshold. In this unit, race does not predict graduation. The hierarchy variables, regardless of measure, also predict graduation in the expected direction. Last Month Maximum Eigenvector Centrality (*β* = -1.46, *se* = 0.81), Last Month Average Eigenvector Centrality (*β* = -6.30, *se* = 2.57), Maximum Eigenvector Centrality (*β* = -1.63, *se* = 0.55), and Average Eigenvector Centrality (*β* = -8.11, *se* = 3.20) were all significant and in the negative direction. The relationships between eigenvector centrality over the entire length of treatment and during the last month and predicted probability of graduation are shown in Fig 4.

**Table 4:** Facility 1, Men’s unit 1: Eigenvector centrality as a predictor of graduation.

	Model 1	Model 2	Model 3	Model 4
Intercept	6.36***	6.42***	6.93***	6.86***
	(1.35)	(1.36)	(1.39)	(1.38)
Age	-0.00	-0.00	-0.01	-0.01
	(0.02)	(0.02)	(0.02)	(0.02)
LSI-R	-0.36***	-0.36***	-0.37***	-0.36***
	(0.04)	(0.04)	(0.04)	(0.04)
Race	0.25	0.24	0.24	0.23
	(0.31)	(0.32)	(0.32)	(0.32)
Days in Program	0.05***	0.05***	0.06***	0.05***
	(0.01)	(0.01)	(0.01)	(0.01)
Last Month Maximum Eigenvector Centrality	-1.64*			
	(0.81)			
Last Month Average Eigenvector Centrality		-6.30*		
		(2.57)		
Maximum Eigenvector Centrality			-1.63**	
			(0.55)	
Average Eigenvector Centrality				-8.11***
				(3.20)
AIC	301.78	299.88	296.92	299.46
BIC	329.97	328.08	325.11	327.66
Log-Likelihood	-144.89	-143.94	-142.46	-143.73
Deviance	289.78	287.88	284.92	287.46
N	812	812	812	812

*** p < 0.001,

** p < 0.01,

* p < 0.05.

**Fig 5:**
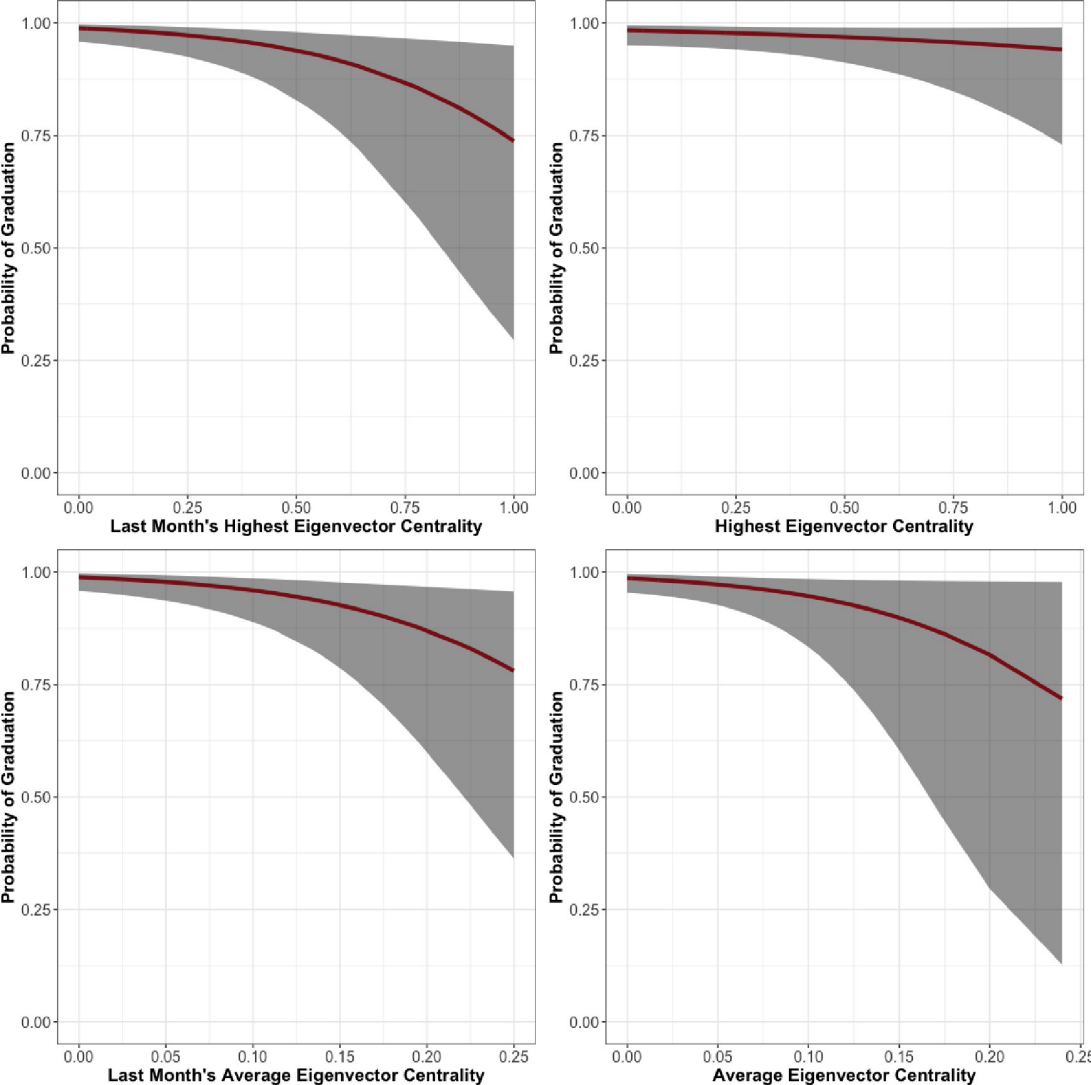
Facility 1, Men’s unit 2 predicted probability of graduation by eigenvector centrality.

In the second Men’s unit of Facility 1, described in Table 5, we see some important differences in the results. First, when significant, the hierarchy measures are significant at a more restrictive threshold than in the first Men’s unit. Second, while the measures examining position during the last month in the hierarchy are significant at p = 0.001 in this unit (Last Month Maximum Eigenvector Centrality, *β* = -3.39, *se* = 1.17; Last Month Average Eigenvector Centrality, *β* = -12.67, *se* = 4.51), the measures that examine position in the hierarchy over a resident’s entire tenure are uncorrelated with graduation. The relationships between eigenvector centrality over the entire length of treatment and during the last month and predicted probability of graduation are shown in Fig 5.

**Table 5:** Facility 1, Men’s unit 2: Eigenvector centrality as a predictor of graduation.

	Model 1	Model 2	Model 3	Model 4
Intercept	2.11	2.07	1.83	2.03
	(2.14)	(2.12)	(2.14)	(2.10)
Age	-0.01	0.00	-0.01	-0.01
	(0.03)	(0.03)	(0.03)	(0.03)
LSI-R	-0.34***	-0.34***	-0.31***	-0.32***
	(0.06)	(0.06)	(0.06)	(0.06)
Race	-0.14	-0.14	0.06	-0.00
	(0.70)	(0.70)	(0.68)	(0.68)
Days in Program	0.08***	0.08***	0.08***	0.08***
	(0.01)	(0.01)	(0.01)	(0.01)
Last Month Maximum Eigenvector Centrality	-3.39***			
	(1.17)			
Last Month Average Eigenvector Centrality		-12.67***		
		(4.51)		
Maximum Eigenvector Centrality			-1.34	
			(1.05)	
Average Eigenvector Centrality				-13.85
				(7.07)
AIC	94.18	94.35	99.73	97.92
BIC	118.05	118.22	123.61	121.79
Log-Likelihood	-41.09	-41.17	-43.87	-42.96
Deviance	82.18	82.35	87.73	85.92
N	395	395	395	395

*** p < 0.001,

** p < 0.01,

* p < 0.05.

**Fig 6:**
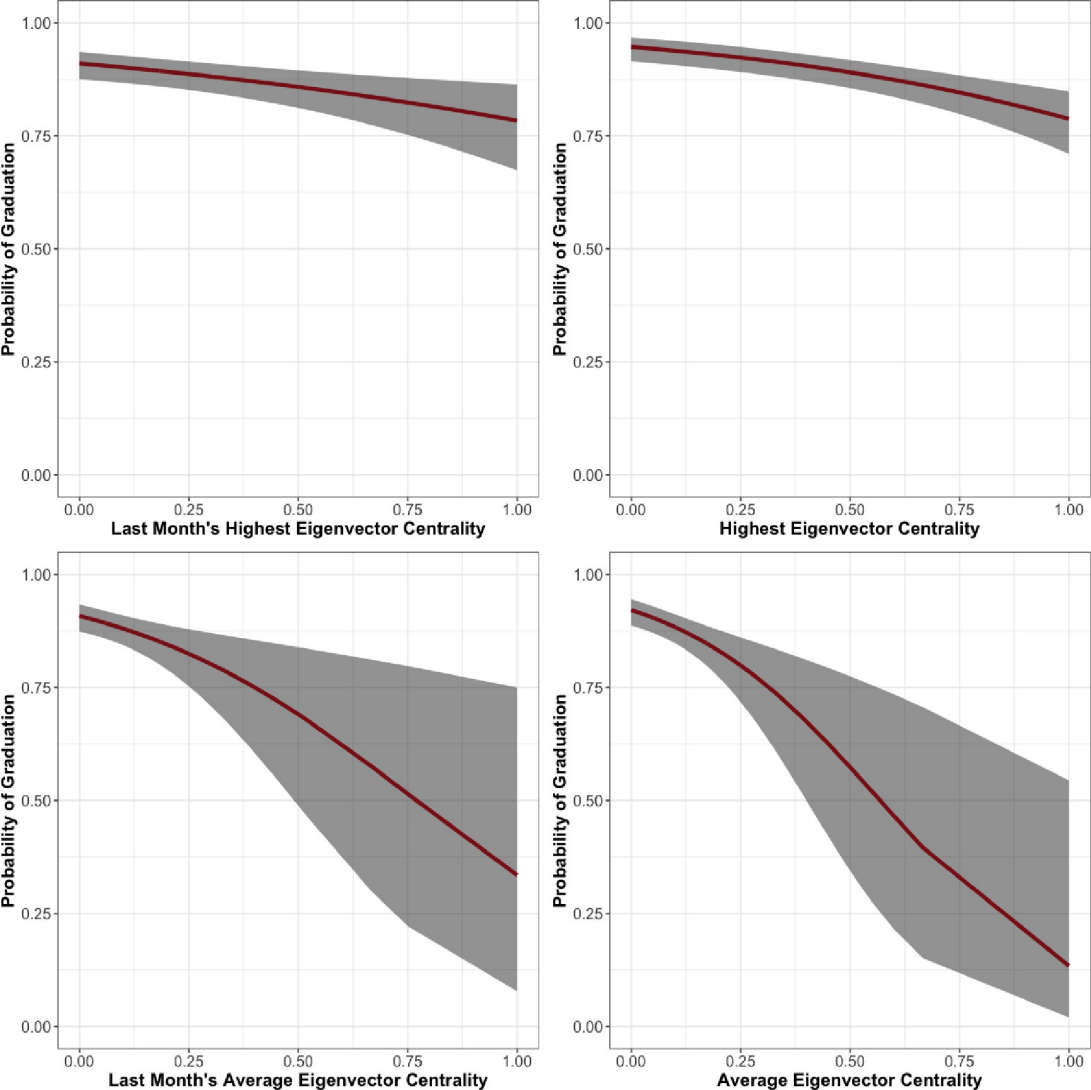
Facility 2 predicted probability of graduation by eigenvector centrality.

The results for Facility 2, which was entirely male, are presented in Table 6. For this unit, LSI-R and time in treatment are significant and in the expected direction. Age is a statistically significant predictor of graduation in three models, in the expected direction. All measures of hierarchy–Last Month Maximum Eigenvector Centrality (*β* = -1.03, *se* = 0.34), Last Month Average Eigenvector Centrality (*β* = -2.98, *se* = 0.99), Maximum Eigenvector Centrality (*β* = -1.57, *se* = 0.34), and Average Eigenvector Centrality (*β* = -4.33, *se* = 1.16)—are statistically significant predictors of graduation. The relationships between eigenvector centrality over the entire length of treatment and during the last month and predicted probability of graduation are shown in Fig 6.

**Table 6:** Facility 2, Men only: Eigenvector centrality as a predictor of graduation.

	Model 1	Model 2	Model 3	Model 4
Intercept	-6.63***	-6.56***	-7.04***	-6.61***
	(1.10)	(1.10)	(1.13)	(1.11)
Age	0.04*	0.04*	0.03	0.03*
	(0.02)	(0.02)	(0.02)	(0.02)
LSI	-0.10***	-0.10***	-0.09***	-0.10***
	(0.02)	(0.02)	(0.02)	(0.02)
Race	0.67	0.69	0.60	0.64
	(0.48)	(0.48)	(0.48)	(0.48)
Days in Program	0.07***	0.07***	0.07***	0.07***
	(0.01)	(0.01)	(0.01)	(0.01)
Last Month Maximum Eigenvector Centrality	-1.03*			
	(0.34)			
Last Month Average Eigenvector Centrality		-2.98*		
		(0.99)		
Maximum Eigenvector Centrality			-1.57***	
			(0.34)	
Average Eigenvector Centrality				-4.33***
				(1.16)
AIC	429.58	429.93	415.24	425.44
BIC	458.58	458.92	444.24	454.43
Log-Likelihood	-208.79	-208.96	-201.62	-206.72
Deviance	417.58	417.93	403.24	413.44
N	928	928	928	928

*** p < 0.001,

** p < 0.01,

* p < 0.05.

**Fig 7:**
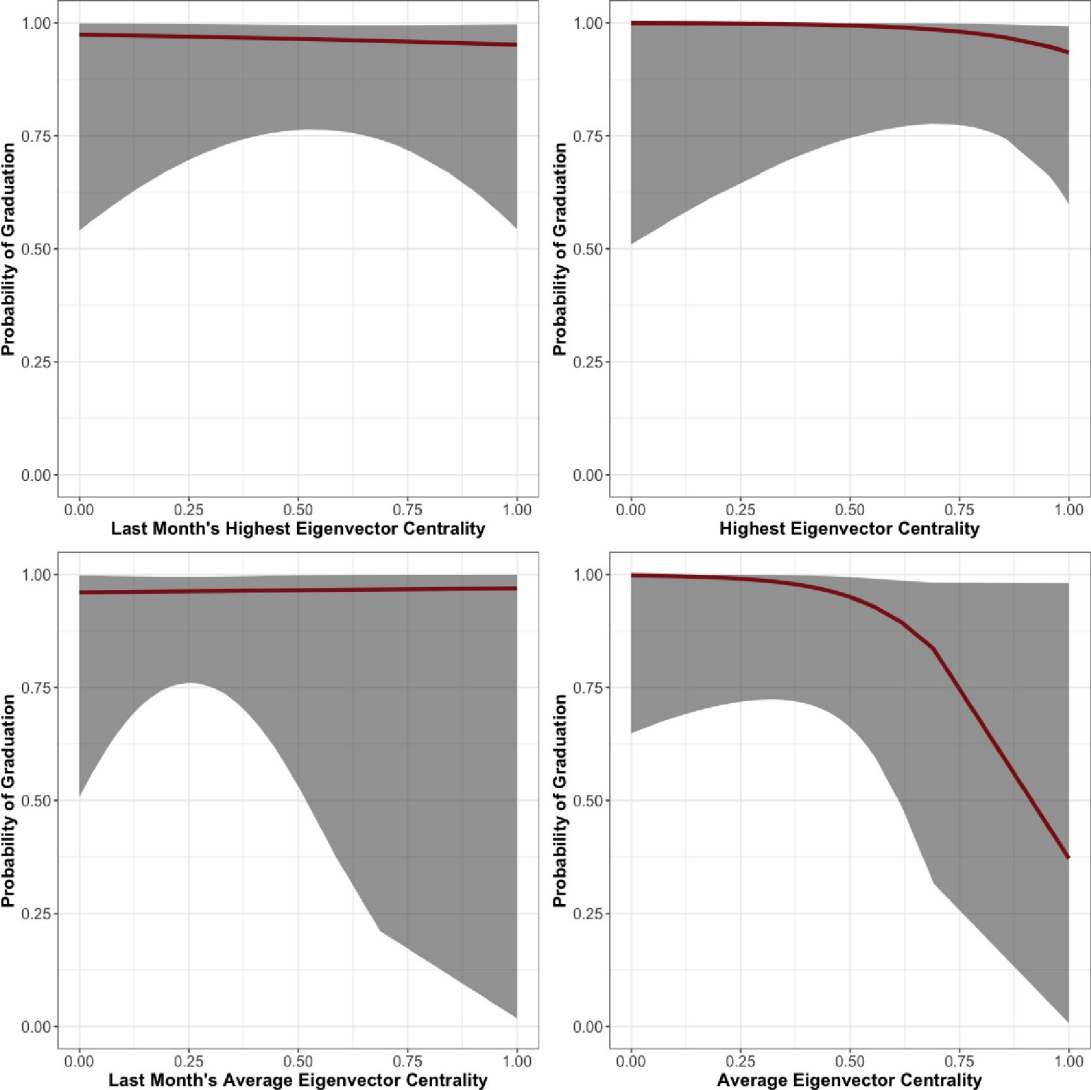
Facility 3, Women’s unit predicted probability of graduation by eigenvector centrality.

In the women’s unit of Facility 3, presented in Table 7, we see departures from the results of prior units. While age and length of time in the program are statistically significant and in the expected direction, none of our hierarchy measures are statistically significant at any conventional threshold. The relationships between eigenvector centrality over the entire length of treatment and during the last month and predicted probability of graduation are shown in Fig 7.

**Table 7:** Facility 3, Women’s unit: Eigenvector centrality as a predictor of graduation.

	Model 1	Model 2	Model 3	Model 4
Intercept	0.18	-0.44	2.48	6.41
	(5.67)	(6.26)	(5.98)	(7.41)
Age	0.01	0.01	0.01	-0.05
	(0.11)	(0.11)	(0.10)	(0.11)
LSI	-0.36*	-0.36*	-0.45*	-0.49*
	(0.15)	(0.16)	(0.20)	(0.22)
Race	1.45	1.48	3.28	2.74
	(2.61)	(2.41)	(3.56)	(3.73)
Days in Program	0.08**	0.08**	0.10**	0.09**
	(0.02)	(0.03)	(0.04)	(0.03)
Last Month Maximum Eigenvector Centrality	-0.66			
	(2.37)			
Last Month Average Eigenvector Centrality		0.27		
		(4.86)		
Maximum Eigenvector Centrality			-5.06	
			(3.97)	
Average Eigenvector Centrality				-6.94
				(4.75)
AIC	29.52	29.59	27.45	26.79
BIC	42.47	42.55	40.41	39.75
Log-Likelihood	-8.76	-8.80	-7.73	-7.40
Deviance	17.52	17.59	15.45	14.79
N	64	64	64	64

*** p < 0.001,

** p < 0.01,

* p < 0.05.

**Fig 8:**
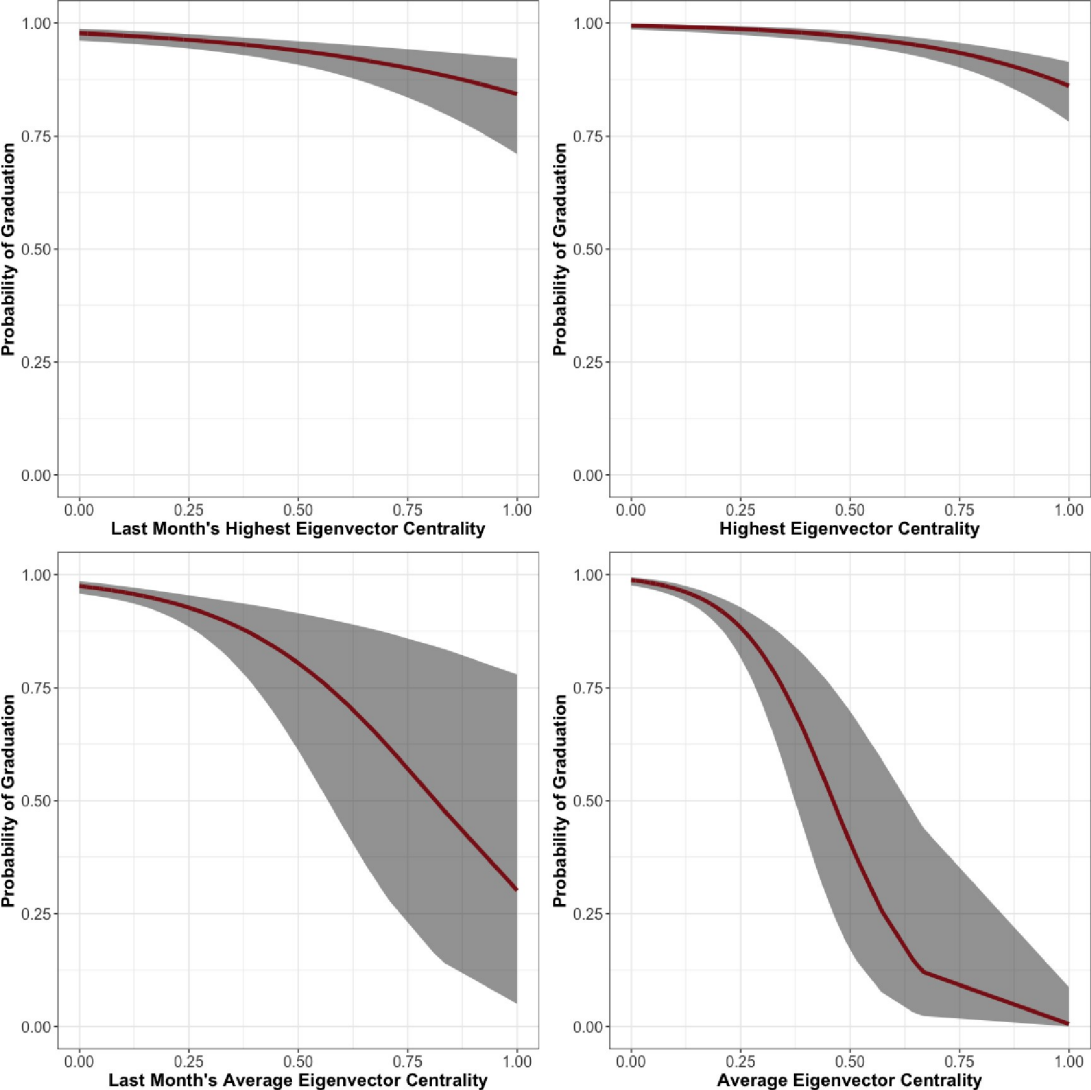
Facility 3, Men’s unit predicted probability of graduation by eigenvector centrality.

Finally, the men’s unit of Facility 3, presented in Table 8 and Fig 6, shows results that we would expect given our hypothesis. LSI and tenure in the program are statistically significant and in the expected direction. In addition, Last Month Maximum Eigenvector Centrality (*β* = -2.10, *se* = 0.54), Last Month Average Eigenvector Centrality (*β* = -4.51, *se* = 1.22), Maximum Eigenvector Centrality (*β* = -3.31, *se* = 0.59), and Average Eigenvector Centrality (*β* = -9.59, *se* = 1.68), all have a statistically significant and negative correlation with graduation. The relationships between eigenvector centrality over the entire length of treatment and during the last month and predicted probability of graduation are shown in Fig 8.

**Table 8:** Facility 3, Men’s unit: Eigenvector centrality as a predictor of graduation.

	Model 1	Model 2	Model 3	Model 4
Intercept	-6.00***	-5.80***	-6.33***	-6.02***
	(1.36)	(1.36)	(1.40)	(1.44)
Age	0.03	0.03	0.03	0.03
	(0.02)	(0.02)	(0.02)	(0.02)
LSI	-0.24***	-0.24***	-0.24***	-0.23***
	(0.03)	(0.03)	(0.03)	(0.03)
Race	0.13	0.06	0.19	0.22
	(0.42)	(0.42)	(0.44)	(0.44)
Days in Program	0.10***	0.10***	0.11***	0.10***
	(0.01)	(0.01)	(0.01)	(0.01)
Last Month Maximum Eigenvector Centrality	-2.10***			
	(0.54)			
Last Month Average Eigenvector Centrality		-4.51***		
		(1.22)		
Maximum Eigenvector Centrality			-3.31***	
			(0.59)	
Average Eigenvector Centrality				-9.58***
				(1.68)
AIC	346.57	347.79	324.19	326.50
BIC	377.73	378.95	355.35	357.66
Log-Likelihood	-167.28	-167.89	-156.09	-157.25
Deviance	334.57	335.79	312.19	314.50
N	1331	1331	1331	1331

*** p < 0.001,

** p < 0.01,

* p < 0.05.

## Discussion

In this study, eigenvector centrality in a social network of peer corrections was used to measure residents’ position in a hierarchy defined by peer corrections. It was assumed that those residents with higher eigenvector centrality were lower in the hierarchy; that is to say, if a resident receives corrections from peers who also receive many corrections, she or he is lower in the hierarchy than those who send the corrections to him or her, who are in turn lower than those who correct them. We hypothesized that residents would rise in the hierarchy over the course of treatment and that those who were lower in the hierarchy were less likely to graduate. We used two measures of eigenvector centrality, mean and maximum, and two time frames, the entire time in treatment and the last month. We expected that eigenvector centrality in the last month would be a better predictor of successful graduation than eigenvector centrality overall.

In all units, mean eigenvector centrality and maximum eigenvector centrality were significantly lower in the last month of treatment. In four of the six units, all measures predicted graduation in the hypothesized direction. In one unit (Facility 1 Men’s Unit 2), only mean and maximum eigenvector centrality in the last month of treatment predicted graduation in the hypothesized direction. In one unit (Facility 3 Women’s Unit), no measure of eigenvector centrality predicted graduation. These latter two units were both somewhat anomalous in the number of individuals participating in the system of corrections. In Facility 1 Men’s Unit 2, the participation rate was lower than in other units, while the Facility 3 Women’s Unit was far and away the smallest of the units (16 beds) and had the fewest total residents over the period during which data was gathered. It may be that it takes a minimum number of residents before eigenvector centrality is a meaningful measure of hierarchy. Last month eigenvector centrality measures were not consistently larger in effect size than overall eigenvector centrality measures. Apparently achieving a higher position in the hierarchy at some point is important to graduation, but that point does not have to be toward the end of treatment.

In no case was eigenvector centrality positively correlated with likelihood of graduation. The change in probability of graduation from those with the lowest eigenvector centrality to those with the highest ranged from somewhat below 25% to well over 50%, depending on the unit. Among the control variables, LSI-R was consistently negatively correlated with successful graduation, while time in treatment was consistently positively correlated with successful graduation (this is true almost by definition, since residents who terminate unsuccessfully often terminate early). The statistical significance of race and age were inconsistent across programs.

These findings suggest that corrections play a considerably more complex role in TC treatment than is immediately obvious. TC clinical theory emphasizes that peer feedback leads to social learning [1, 2, 36, 37]. However, in these TCs, peer corrections form a hierarchy through which residents move; in five of these six TCs, movement through the hierarchy predicts successful graduation. The peer hierarchy is therefore more than a mechanism for delivering feedback. It allows residents the chance to grow, learn and experience success by moving through the network. Previous findings show that peer relationships are critical to success in the TC [16, 17], but this study demonstrates that the dynamics of change in those relationships is also important for treatment success.

This analysis adds to the growing body of evidence that success in substance abuse treatment is not only a function of the individuals to whom one is directly connected [38] but also of more complex social network structures and peers several links away in the network [12, 18, 39, 40]. The idea that network structure beyond direct social support matters for sobriety is consistent with the broader social network literature [41–43]. Since eigenvector centrality is a function of the number of connections which an individual’s direct connections have, successful graduation is influenced by peers at least two links away in the network. This is likely to be a very large percentage of the total number of peers in any TC; the community as a whole is, in fact, engaged in the treatment of any one individual [1]. More broadly, if an individual’s sobriety is influenced by peers several links away in the network, then the deep network that comes with connection to a group of self-consciously sober peers—such as can be found in TCs, sober living houses, or self-help groups—is likely to be of more value than simply having friendships with disconnected individuals who do not drink or use drugs.

In TC clinical theory the importance of the entire network is summed up in the injunction that the community as a whole is the method of treatment [1]. The relationship of the individual to the community is seen as changing over time. Early in treatment the resident is primarily concerned with him or herself, with concern slowly expanding to the entire community. Residents whose concern does expand to the entire community are seen as having better outcomes [1]. Movement up a hierarchy based on decreasing eigenvector centrality accomplishes this clinical sequence, as the resident changes her or his role from being the focus of feedback to giving feedback to those who in turn give it to others.

The complexity of the network makes it hard to imagine that staff members intervene to organize it. Program phase and work positions attained may serve as signals, but time in program should serve as a proxy control for these variables. It is therefore likely that hierarchy is self-organizing, emerging from the interactions of the residents [44, 45]. A self-organizing network hierarchy based on eigenvalue centrality helps to address several problems in any TC. Residents who are more central receive feedback from peers who are themselves receiving feedback, encouraging information flow through the network. This structure, along with the ability of staff to monitor written corrections and the public nature of the corrections, means that residents are unlikely to receive counterproductive feedback. Feedback from multiple peers who live and work together is a form of swarm intelligence [46] in which knowledge distributed through the network is applied to the problem of recovery. Such a network can effectively individualize treatment within the TC structure, even for a large unit. As long as residents progress through the hierarchy, graduates will be replaced as role models, and their graduation will not unduly disrupt the feedback system.

## Conclusion

This study only draws on data from six units in three Midwestern TCs. This clearly limits external validity, particularly since there can be considerable variation between facilities and even groups within a given facility [47]. Moreover, if the hierarchy is self-organizing, staff may have limited means of influencing the structure directly, and so the study does not necessarily point the way to clinical network interventions.

Nevertheless, these findings have clinical implications, because staff can make choices that foster the resident feedback network even if they do not directly influence it. Several of these are already in the literature in one way or another; for instance, an orderly unit atmosphere [16, 47] seems likely to foster a resident hierarchy, and in turn benefit from it. It is likely to be valuable for staff, in their own position as rational authorities [1], to either formally or informally continue to educate residents on TC norms, so that the feedback that moves through the social network is of value. It should also be possible to consciously teach residents how to give feedback to peers [2]. This study also implies that all residents should participate in the peer feedback system; the two units with the fewest participants in the feedback network also showed the weakest relationship between progress through the hierarchy and graduation.

Finally, the clinical logic of peer feedback in TCs needs to incorporate both social learning (1) and other uses residents make of it. In the case of corrections, peers use them to organize an informal hierarchy in addition to social learning. Interventions that are based on mutual aid by definition allow clients to take partial responsibility for implementation, and they may use specific aspects of the intervention in multiple ways. It is therefore important to expand on the current literature documenting the way in which TC residents, and participants in other mutual aid based programs, view and make use of clinical tools (5, 6, 20). Research understanding of TCs will remain incomplete until we incorporate the understanding of TC residents have of their own participation.
